# Structural basis of GC-1 selectivity for thyroid hormone receptor isoforms

**DOI:** 10.1186/1472-6807-8-8

**Published:** 2008-01-31

**Authors:** Lucas Bleicher, Ricardo Aparicio, Fabio M Nunes, Leandro Martinez, Sandra M Gomes Dias, Ana Carolina Migliorini Figueira, Maria Auxiliadora Morim Santos, Walter H Venturelli, Rosangela da Silva, Paulo Marcos Donate, Francisco AR Neves, Luiz A Simeoni, John D Baxter, Paul Webb, Munir S Skaf, Igor Polikarpov

**Affiliations:** 1 Instituto de Física de São Carlos, Universidade de São Paulo, Avenida Trabalhador São Carlense, 400 CEP 13560-970 São Carlos, SP, Brazil; 2 Instituto de Química, Universidade Estadual de Campinas, Caixa Postal 6154, 13084-862 Campinas, SP, Brazil; 3 C3-137, Molecular Medicine Department, College of Veterinary Medicine, Cornell University, Ithaca, NY, ZIP 14853, USA; 4 Departamento de Química, Faculdade de Filosofia, Ciências e Letras de Ribeirão Preto, Universidade de São Paulo, Avenida Bandeirantes n. 3900, 14040-901, Ribeirão Preto, SP. Brazil; 5 Núcleo de Pesquisa em Ciências Exatas e Tecnológicas, Universidade de Franca, Avenida Dr. Arnaldo Salles de Oliveira, 2001, 14404-600, Franca, SP, Brazil; 6 Departamento de Ciências Farmacêuticas, Universidade de Brasília, Brasília, DF, 70900-910l, Brazil; 7 Diabetes Center, Metabolic Research Unit, and the Department of Medicine, University of California San Francisco, San Francisco CA, 94143, USA

## Abstract

**Background:**

Thyroid receptors, TRα and TRβ, are involved in important physiological functions such as metabolism, cholesterol level and heart activities. Whereas metabolism increase and cholesterol level lowering could be achieved by TRβ isoform activation, TRα activation affects heart rates. Therefore, β-selective thyromimetics have been developed as promising drug-candidates for treatment of obesity and elevated cholesterol level. GC-1 [3,5-dimethyl-4-(4'-hydroxy-3'-isopropylbenzyl)-phenoxy acetic acid] has ability to lower LDL cholesterol with 600- to 1400-fold more potency and approximately two- to threefold more efficacy than atorvastatin (Lipitor^©^) in studies in rats, mice and monkeys.

**Results:**

To investigate GC-1 specificity, we solved crystal structures and performed molecular dynamics simulations of both isoforms complexed with GC-1. Crystal structures reveal that, in TRα Arg228 is observed in multiple conformations, an effect triggered by the differences in the interactions between GC-1 and Ser277 or the corresponding asparagine (Asn331) of TRβ. The corresponding Arg282 of TRβ is observed in only one single stable conformation, interacting effectively with the ligand. Molecular dynamics support this model: our simulations show that the multiple conformations can be observed for the Arg228 in TRα, in which the ligand interacts either strongly with the ligand or with the Ser277 residue. In contrast, a single stable Arg282 conformation is observed for TRβ, in which it strongly interacts with both GC-1 and the Asn331.

**Conclusion:**

Our analysis suggests that the key factors for GC-1 selectivity are the presence of an oxyacetic acid ester oxygen and the absence of the amino group relative to T_3_. These results shed light into the β-selectivity of GC-1 and may assist the development of new compounds with potential as drug candidates to the treatment of hypercholesterolemia and obesity.

## Background

Thyroid hormones (TH) have important roles in development and homeostasis. Thyroid receptor (TR) activation occurs when an agonist ligand such as TH or similar compounds binds to a hydrophobic pocket in the core of its ligand-binding domain (LBD). This causes conformational changes which allow DNA bound receptors to interact with coactivators, mediating transcription. TH has a considerable potency as a cholesterol reducer in serum, and stimulates basal metabolic rate, promoting weight loss by the increase of thermogenesis [1,2]. However, deleterious side effects such as tachycardia and atrial arrhythmia are well-documented rendering TH treatment unviable for the treatment of obesity and hypercholesterolemia [3-8].

While TH cannot be used to treat dyslipidemias and obesity, TR isoform selective agonists may counteract these problems. The human (h) TR exists in two isoforms, hTRα (NR1A1), and hTRβ (NR1A2). hTRα is expressed at higher levels in heart and plays a major role in regulation of heart rate, whereas TRβ is the predominant isoform that is expressed in the liver and pituitary and is involved with hepatic cholesterol metabolism and regulation of metabolic rate [9,10]. Evidence from human patients and mouse models suggests that elevated cholesterol and body weight can be decreased by the preferential activation of hTRβ *versus *hTRα. Since obesity and atherosclerosis are important medical problems with impact on morbidity and mortality, development of β-selective thyromimetics and improved understanding of their actions is an important pharmacological and biomedical issue.

Similar to other nuclear receptors, TRs exhibit a modular structure, composed of functionally separable domains. The most highly conserved domains are the LBD and DNA binding domain (DBD), with the former responsible for multiple activities, including hormone binding, homo and/or heterodimerization, molecular interactions with heat-shock-proteins, and transcriptional activation and repression [11-14]. All of these properties are regulated by ligand-dependent conformational changes [13,14]. Therefore, improved understanding of the three-dimensional structure of a liganded LBD and dynamic changes that occur on ligand binding are critical for understanding hormone action. While hTRβ LBD crystal structures of complexes with natural hormones (T_3_, thyroxine T_4_, TRIAC) and synthetic ligands (among them GC-1, GC-24, KB-141) are available, structural information on hTRα LBD is scarce [15-19]. Even less information about the role of TR flexibility and dynamics in ligand binding is available. For example, it was only recognized recently that TR ligands dissociation (and, presumably, association) can proceed via multiple escape pathways that may be ligand specific [20,21].

**Figure 1:**
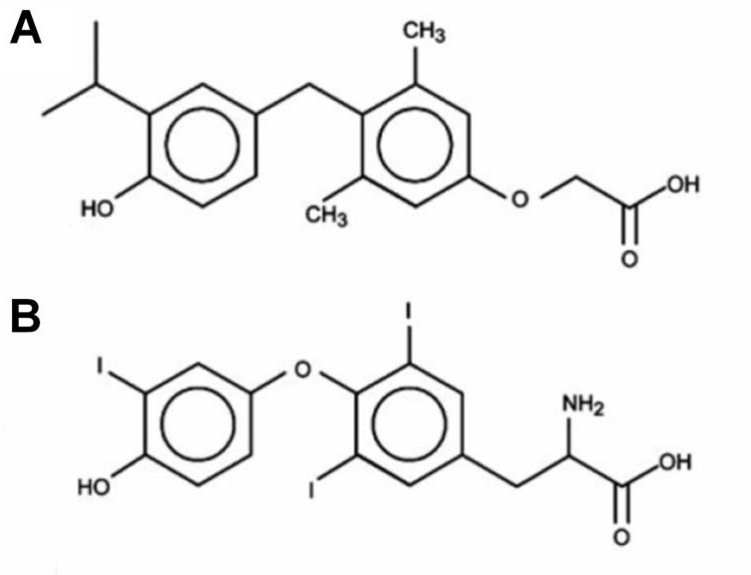
**Bleicher1.png**. Chemical formulas of GC-1 ***(a) ***and T_3 _***(b)***.

GC-1 (3,5-Dimethyl-4-(4'-hydroxy-3'-isopropylbenzyl)-phenoxy acetic acid; Fig. 1), is an analogue of the thyromimetic DIMIT that shows comparable affinity for hTRβ_1 _to T_3 _and desirable isoform selectivity, binding to hTRα_1 _with a 10-fold lower affinity [22,23]. GC-1 shows extremely encouraging results in animal models [24-31]. GC-1 lowers cholesterol in hypothyroid mice and monkeys at concentrations that do not affect heart rate and lowers LDL cholesterol with 600- to 1400-fold more potency and approximately two- to threefold more efficacy than atorvastatin (Lipitor^©^)[24,28,29]. GC-1 also decreases plasma levels of other atherogenic lipids; triglycerides and lipoprotein (a). Further, GC-1 induces loss of body fat with no detectable loss of muscle [28,29]. Finally, GC-1 stimulates important steps in reverse cholesterol transport [31]. GC-1 is also a synthetically accessible scaffold that serves as a platform for the development of new TR agonists and antagonists [32-34]. Thus, improved understanding of the structural basis of GC-1 TR binding and selectivity is of paramount importance for the rational design of thyromimetics.

Presently, only an X-ray structure of TRβ in complex with GC-1 is available. While this structure reveals extensive hydrogen bond formation between the carboxylate tail of the ligand and polar residues in the hormone binding pocket, improved understanding of the molecular basis of isoform-selective binding awaits direct comparisons of structures of both TR isoforms in complex with this ligand [16]. Here, we report the new structures of hTRα and hTRβ LBDs in complex with GC-1 and T_3 _and results of MD simulations performed with these structural models. Our data suggests that most of the selective binding of GC-1 is related to alternate conformations of a conserved Arg residue in the pocket (Arg228α/Arg282β) that, in turn, is influenced by selective interactions of this Arg residue with the single isoform-selective residue in the pocket.

## Results

### X-ray structures

Cell-based assays confirmed that our preparation of GC-1 (Materials and Methods) has potent TR agonist activity, with maximum efficacy similar to that of T_3 _and selectivity for TRβ vs. TRα (Additional file 1, Fig. 1S). Co-crystallization of GC-1 with both hTRα and hTRβ yielded structures with good geometric and crystallographic parameters (Table 1). The Ramachandran plots are acceptable for both structures. These plots assess the main chain configuration of a protein by a 2D graph displaying the two torsional angles (ϕ and ψ) of each amino acid residue, which should be clustered in regions that don't cause steric clashes between them. No residues were observed in the disallowed regions of the plots, except for Arg188 in TRα structure in P2_1_2_1_2_1_. Electron density for the ligand was clear and well-defined in all the structures obtained.

**Table 1:** Crystallographic information

	hTRα + GC-1 (first crystal form)	hTRα + GC-1 (second crystal form)	hTRα + T3	hTRβ + GC-1	hTRβ + T3
Space group	P2_1_2_1_2_1_	C2	P2_1_2_1_2_1_	P3_1_21	P3_1_21
Cell Parameters (Å)	a = 59.91 b = 80.35 c = 102.89	a = 89.80 b = 78.78 c = 43.07 β = 95.18	a = 59.98 b = 80.79 c = 102.21	a = 68.99 b = 68.99 c = 130.86	a = 68.95 b = 68.95 c = 131.11
Resolution (Å)	43.44 – 1.85	58.72 – 2.50	63.25 – 1.87	33.35 – 2.55	33.35 – 2.30
I/(σ)	35.81 (3.64)	7.8 (2.1)	7.8 (2.0)	17.66(2.31)	25.75(2.02)
Total number of reflections	40931	9551	39069	11291	15596
Completeness (%)	100	91.7 (83.7)	98.7 (98.9)	96.9(99.0)	99.0
Multiplicity	6.3 (6.0)	7.2 (7.2)	8.2 (7.8)	3.9 (3.7)	16.1 (5.5)
R_merge_^a ^(%)	4.3 (49.2)	7.6 (36.6)	5.9 (38.3)	6.7 (57.7)	7.7 (56.5)
Bond length (Å)	0.037	0.018	0.041	0.033	0.024
Bond angles (deg)	3.047	1.813	3.846	3.082	2.631
R_factor _(%)	14.9	18.8	14.8	20.3	20.4
R_free _(%)	18.8	26.3	18.6	27.6	25.4

### Ligand binding pocket

**Figure 2:**
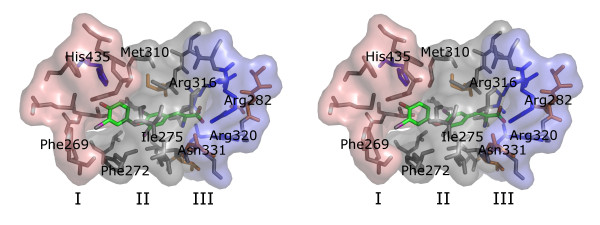
**Bleicher2.png**. The TR hormone binding site, shown as T_3 _bound to hTRβ. The regions shown in red, gray and blue correspond to regions I, II and III described in the text. Apolar residues are shown in gray, basic residues in blue (namely His435, Arg316, Arg282 and Arg320) and polar residues in orange (including Asn331).

The TR ligand binding pocket is roughly subdivided in three regions, as shown in Figure 2. Region I is largely hydrophobic but contains a single polar residue (His435β/381α) which forms a hydrogen bond interaction with a hydroxyl group of most thyromimetics or thyroid hormones, including T_3_, T_4 _and TRIAC. TH analogues synthesized without the phenol on that position tend to lack functionality [18]. Region II is mainly composed of apolar residues that interact with the thyronine rings of TH derivatives or phenolic rings of thyromimetics such as GC-1. Region III, occupied by the oxyacetic acid moiety of GC-1, is mostly polar and is the likely location in which changes in interaction with T_3 _and GC-1 determine the selectivity of the compound because it harbours the only subtype selective residue in the hTRα and hTRβ binding pockets; hTRα has a Ser 277 residue, which is substituted by Asn 331 in hTRβ. GC-1 carboxyl group interactions are mostly mediated by three arginine residues (Arg228, Arg262 and Arg266 in hTRα) through water molecules supported hydrogen bonding network.

**Figure 3:**
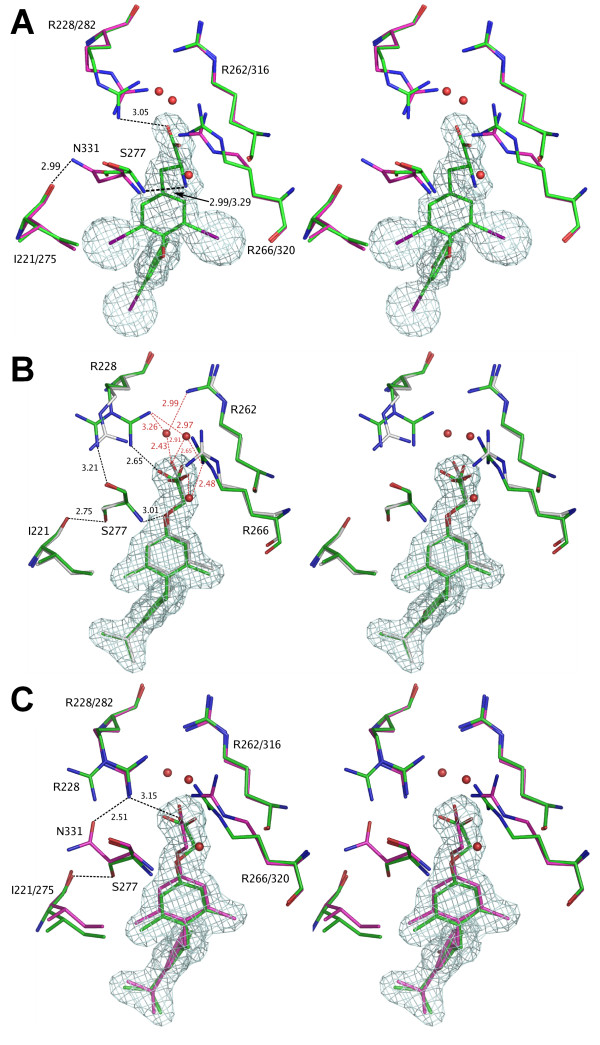
**Bleicher3.png**. Ligand-receptor interactions for thyroid receptors: ***(a) ***T_3 _as bound to hTRα (green) and hTRβ (magenta). All interactions are maintained between the ligand and the binding site residues in both hTR isoforms. Both Ser277 and Asn331 interact with the amino group of T_3 _through their amide nitrogen, leading to similar conformations of these residues. ***(b) ***GC1 bound to TRα: multiple conformations of the Arg228 are observed. In the productive conformation there is a strong interaction with the ligand (cyan), while in non-productive conformations this residue interacts with the side-chain of Ser277. The Arg228 double conformation is observed in the first crystal form of hTRα LBD+GC1 complex (Table 1). In the intermediate conformation Arg228 interacts both with the GC1 and the Ser277 amino group (white, second hTRα LBD+GC-1 crystal form, Table 1). ***(c) ***Comparison of GC1 bound to hTRα and hTRβ. For hTRβ (magenta) only a single productive conformation of the Arg282 side-chain was observed, which resembles the productive Arg228 (hTRα) conformation (green). Arg282 (hTRβ), is strongly interacting with the ligand and its productive conformation is locked in place by the interactions with the side-chain of Asn331.

Comparison of hTRα and hTRβ complexed with T_3 _confirms that there is a similar binding mode [35,36]. The hormone binds both receptors in the extended conformation forming the same interactions with the amino acid residues of the active sites (Fig. 3a). The lack of structural differences is consistent with the fact that T_3 _binds both TR isoforms with similar affinity.

By contrast, the binding modes of GC-1 are clearly different. Similar to T_3_, GC-1 binds both TRs through a combination of hydrophobic interactions and two hydrogen bond sites. The first hydrogen bond involves the phenolic hydroxyl of the outer thyronine ring and a histidine residue in region I of the pocket (His381/435). The second site involves the GC-1 oxyacetic acid substituent and the positively charged pocket formed by three arginine residues in region III, Arg228/282, Arg262/316 and Arg266/320 (Fig. 3b and 3c). In hTRβ, Arg282 from helix 3, and Arg316 and Arg320 from helix 6 interact with the GC-1 oxyacetic acid carboxylate both directly and via water-mediated hydrogen bond interactions, as previously observed in a previous hTRβ/GC-1 structure [16]. Asn331 forms a hydrogen bond with Arg282 that anchors the latter in place and permits it to form a stable interaction with the GC-1 carboxylate group.

In hTRα, a different picture arises. First, the GC-1 oxyacetic acid ester oxygen forms a hydrogen bond with the main chain amino group of TRα specific Ser277 residue. The Ser277 side chain hydroxyl participates in a hydrogen bond interaction with the Ile221 main chain carbonyl group, which is not available to Asn331 in hTRβ. This means that Ser277 fails to engage in a hydrogen bond contact with Arg228 analogous to that observed between Asn331 and Arg282. Thus, Arg228 is not anchored by contacts with the subtype specific residue. In addition, Ser277 is displaced by 1.64 Å toward the ligand compared to the T_3_-bound hTRα[36]. This effect, coupled with the fact that dislocation and the smaller size of Ser relative to Asp in TRβ provides room for the Arg228 side chain to move, permits Arg228 to adopt apparent multiple conformations, as described below.

To distinguish between the conformations of the Arg228 side chain, we will call the first, similar to the TRβ +GC-1 structure, the *productive conformation*, and the two alternatives, the *non-productive conformations*. Arg228 displays a double conformation in the first hTRα crystal form (P2_1_2_1_2_1_, Table 1): a productive conformation identical to that of hTRβ and a non-productive conformation in which it points away from ligand (Fig. 3a, in green). In the second crystal form (C2 space group, Table 1) Arg 228 is in another non-productive conformation (Fig. 3a, in white) which is an intermediate between two conformations of the same residue found in the first crystal form. Differential positioning of the Arg228 side chain is related to hydrogen bond with the Ser277 carbonyl group that flips the Arg228 side chain into an alternative conformation (Fig. 3c). To compensate this conformational change, a water molecule (W343) bridges the Arg228 side chain and the GC-1 carboxylate group. This weakens direct interaction with Arg228 and forces GC-1 into a new conformation which involves re-orientation of its carboxyl group.

Thus, our structures reveal different binding modes for GC-1 in TRα and TRβ. In TRβ a single productive conformation is observed, in which the ligand interacts strongly with Arg282, while this residue interacts with the carbonyl group of Asn331. In TRα, multiple conformations are observed. In the productive conformation the ligand interacts strongly with the Arg228 residue and with the Ser277 by its oxyacetic oxygen. In the non-productive conformations GC-1 maintains the interaction with Ser277 but the strong interaction of GC-1 with Arg228 is lost, giving way to a hydrogen bond of this residue with the main carbonyl of Ser277.

### Molecular dynamics simulations

**Figure 4:**
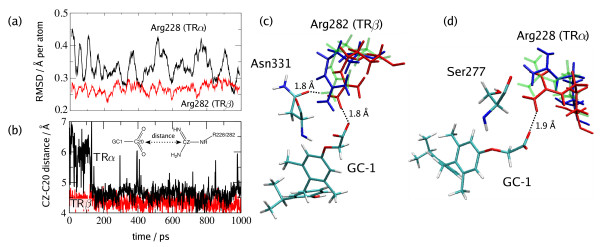
**Bleicher4.png**. Conformational variability of the Arg228 (hTRα) and Arg282 (hTRβ) residues as observed from molecular dynamics simulations. ***(a) ***The greater flexibility of the Arg228 side chain relative to the Arg282 side chain can be observed by the larger RMS deviations. This flexiblity results from weaker anchoring of this side chain in hTRα. ***(b) ***Two binding modes can be distinguished if one computes the CZ-C20 distance. ***(c) ***The snapshots of Arg282 show practically the same productive conformation, locked in place by the strong interaction with the Asn331 side-chain. ***(d) ***Asn331 (hTRβ) to Ser277 (hTRα) substitution removes these conformational restrains and allows Arg228 to sample a much wider range of conformations. The non-productive conformations encountered in the simulations resemble closely the non-productive conformations of Arg228 found in the crystallographic structures.

Conformational variability of the Arg228 residue in TRα was also observed in MD simulations (Fig. 4). For hTRβ the residue oscillates around a mean defined value, indicating a single conformation. In hTRα, the RMSD of the Arg228 side-chain has much larger flexibility, as shown in Figure 4(a). This is a result of the weaker anchoring it displays relative to the Arg282 of TRβ that interacts both with the ligand and with the Asn331 residue. This weak anchoring of the Arg228 residue allows for multiple binding modes, which are characterized by basically two different distances between the carboxylate of GC1 and Arg228, that can be seen in Figure 4(b). Overlaps of snapshots of the simulations at different times in Figure 4(c) and 4(d) illustrate the conformations and the binding modes observed.

**Figure 5:**
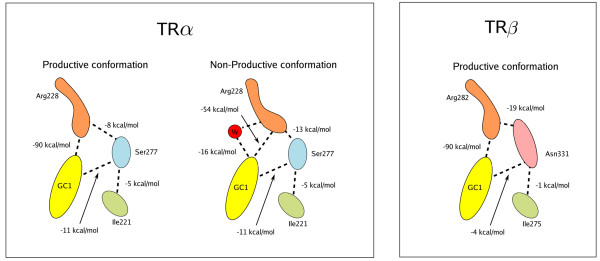
**Bleicher5.png**. Average interaction energies involved in the binding of GC-1 to TRα and TRβ as computed from molecular dynamics simulations.

To understand whether the apparent instability of the GC-1-Arg228α explains weaker ligand binding to this receptor isoform, we analyzed the energies of these interactions obtained from the molecular dynamics simulations. Figure 5 shows a schematic representation of the binding modes of GC-1 in hTRα and hTRβ, together with the average energies of each interaction as computed by MD simulations. In the productive conformations of GC-1 bound both to hTRα and hTRβ there is a strong (-90 kcal/mol) interaction between the GC-1 carboxylate and Arg228/282. The second strongest interaction is the hydrogen bond that Arg282 forms with the Asn331 residue in the hTRβ isoform (-19 kcal/mol). The corresponding Arg228-Ser277 interaction is comparatively weak (-8 kcal/mol). In the non-productive conformation observed in hTRα, the GC-1-Arg228 interaction is weakened to -54 kcal/mol. This conformational change is accompanied by the entrance of a water molecule that forms a hydrogen bond with the carboxylate of GC-1. Also relevant is the fact that the Arg228 residue in the non-productive conformations strengthens its interaction with Ser277 (from -8 to -13 kcal/mol). The Ser277-Ile221 interaction does not appear to be particularly relevant. The hydrogen bond of GC-1 with the Ser277 is quite strong (-11 kcal/mol), and somewhat favours binding to hTRα relative to hTRβ. A slight rearrangement of the hydrophobic residues in the binding site is also observed, but the differences in interaction energies are negligible (< 1 kcal/mol).

Together, these data suggest a plausible explanation for the greater affinity of GC-1 for the hTRβ isoform. The productive conformations of the Arg228/282 residues are the driving forces leading to ligand binding stabilization. Very strong interactions of these residues with GC-1 appear in productive conformations of both TR-isoforms. The reason for the productive conformation to be more stable in hTRβ than in hTRα is related to the differences in the side-chains of the Ser277 and Asn331 residues. In hTRβ, Arg282 interacts, at the same time, with the carboxylate of the ligand and with the side chain of Asn331, which locks in place the productive conformation of the former residue. In hTRα, Arg228 has the potential to engage in strong charge-charge interactions with the ligand, but the short side-chain of Ser277 residue is unable to lock it in place. Therefore, the stabilization of the productive conformation resulting from the Arg282-Asn331 interaction is of key importance for hTRβ selectivity of GC-1.

### Specific Binding of GC-1 is related to the Absence of the Amino Group

**Figure 6:**
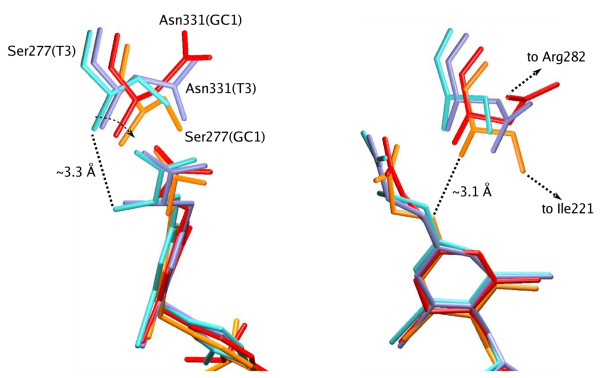
**Bleicher6.png**. Superposition of the crystal structures of T_3 _(blue and sky blue) and GC1 (red and orange) bound to two hTR isoforms highlights the conformational variability associated with the Ser277 and Asn331 residues. This variability is mostly a result of the lack of the amine group in GC-1, but its presence in T_3_.

Why is this isoform-specific binding mode available to GC-1 and not T_3_? T_3 _contains an amino group that is missing from GC-1 and it is known that removal of the amino group in both T_3 _and GC-1 analogues improves β-selectivity. For T_3_, the removal of the amino group results in a slightly β-selective ligand [17]. For GC-1 analogues it turns the DIMIT ligand (1.6 times selective) into the propionic-acid GC-1, which is 4.5 times selective towards TRβ^22^. Superposition of structures of T_3 _and GC-1 bound to both TR isoforms reveals significant movement of the part of the TR polypeptide backbone containing the Ser277 and Asn331 residues in the GC-1 structures (Fig. 6), that is a result of the absence of the T_3 _amino-Ser277/Asn331 interactions. This movement allows the Ser277 and Arg331 residues to interact with other residues of the binding pocket (Ile221 with Ser277 and Arg282 with Arg331) in the presence of GC-1. Hence, removal of the T_3 _amino group allows for the displacement of the chains containing the Ser277 and Asn331 residues and resulting difference in binding modes; as described above, the Asn331-Arg282 (hTRβ) interaction stabilizes the productive conformation, while the Ser277-Ile221 (hTRα) interaction has no effect on ligand affinity.

## Discussion

We have presented a structural investigation of ligand binding modes of the two isoforms of human TR with T_3 _and GC-1 and a computational analysis of the interaction energies involved in these systems. Our findings suggest that the basis of GC-1 selectivity lies in the differences in ligand and protein conformations, which are related to the absence of the amino group relative to T3 and to the Ser/Asn active-site substitution. This substitution increases the space available to a conserved Arg residue (Arg228) permitting it to adopt multiple conformations that are indicative of increased flexibility. While hTRβ Arg282 binds to the ligand in a single conformation, hTRα Arg228 has the potential to flip away from the ligand in non-productive conformations, resulting in loss of an important interaction for the stabilization the ligand.

Our model is supported by MD simulations. The simulations reveal that, for GC-1 bound hTRα, Arg228 can assume multiple conformations, and although GC-1 has the additional interaction with Ser277, the overall binding energy of hTRα is smaller. On the other hand, the productive conformation of Arg282 with GC-1 in hTRβ is very stable both in X-ray structure and in MD simulations, being locked in place by the strong interaction of the side-chain of Asn331.

## Conclusion

The x-ray structures of both thyroid hormone receptor isoforms as bound to GC-1 and to natural hormone T3 permitted the proposal of a β-selectivity mechanism for GC-1, which is supported by molecular dynamics simulations of the complexes.

The major contributions for selectivity in GC-1 are the presence of an oxyacetic acid ester oxygen and the absence of the amino group relative to T_3_. These features favour different conformations for residues in the binding site which, as described, modify the binding network of the ligand for the two isoforms.

In summary, our analysis sheds light into the molecular basis of TRβ-selectivity of GC-1 and may assist rational design of new TH analogs with improved TRβ selectivity lacking the undesirable properties of the natural hormones.

## Methods

### Synthesis of GC-1

GC-1 (compound **7**) was synthesized from commercial starting materials by the convergent synthetic route (Additional file 1, Fig. 2S), as described previously by Chiellini *et al. *[37].

The bromo-ether **1**, prepared from the commercial 4-bromo-2-isopropylphenol, was reacted with the protected aldehyde **3**, prepared from the commercial 4-bromo-3,5-dimethylphenol, to produce the biaryl alcohol **4**. Hydrogenolysis of alcohol **4 **gives the diether **5**, which was treated with TBAF (tetra-*n*-butylammonium fluoride) to provide the selective removal of the phenolic silyl ether proteting group, furnishing the free phenol **6**. Alkylation of **6 **with bromoacetic acid, followed by removal of the methoxymethyl phenolic protecting group under acid conditions, produced the target compound GC-1 (**7**), in 39% overall yield.

### 4-Bromo-2-isopropyl phenyl methoxymethyl ether (1)

Monochloromethylether (1.55 mL, 19.2 mol) was added dropwise to a solution of 4-bromo-2-isopropylphenol (2.10 g, 9.62 mmol) and diisopropylamine (3.35 mL, 19.3 mmol) in THF (10 mL) under inert atmosphere and stirred at room temperature during 30 min. The reaction mixture was diluted with 30 mL of water and extracted with dichloromethane (4 × 5 mL). The organic layer was dried over anhydrous MgSO_4_, filtered, and evaporated under reduced pressure to give an oil, which was purified by column chromatography through silica gel, using *n*-hexane:ethyl acetate (95:5) as eluent, to give the pure product **1 **(2.20 g, 8.50 mmol, 88%). ^1^H NMR (CDCl_3_, 400 MHz) δ 1,20 (d, 6H, *J *= 6,9 Hz), 3,31 (heptet, 1H, *J *= 7,1 Hz), 3,51 (s, 3H), 5,20 (s, 2H), 6,95 (d, 1H, *J *= 8,7 Hz), 7,22 (dd, 1H, *J *= 8,7 and 3.1 Hz), 7,40 (d, 1H, *J *= 3,1 Hz); ^13^C NMR (CDCl_3_, 100 MHz) δ 22.7, 27.0, 56.1, 95.0, 114.5, 116.1, 129.8, 140.1, 154.5.

### *O*-Triisopropylsilyl-4-bromo-3,5-dimethylphenol (2)

A solution of 4-bromo-3,5-dimethylphenol (1.50 g, 7.45 mmol), imidazole (1.26 g, 16.3 mmol) and triisopropylsilyl chloride (1.36 g, 7.1 mmol) in dichloromethane (30 mL) was stirred for 1 h at room temperature. The reaction mixture was diluted with dichloromethane (20 mL), washed with water (10 mL), brine (10 mL), dried over anhydrous MgSO_4_, filtered and evaporated to give an oil, which was purified by column chromatography through silica gel, using *n*-hexane:ethyl acetate (90:10) as eluent, to furnish compound **2 **(1.95 g, 6.70 mmol, 89%) as an oil. ^1^H NMR (CDCl_3_, 400 MHz) δ 1.15 (d, 18H, *J *= 6.9 Hz), 1.26 (m, 3H), 2.35 (s, 6H), 6.60 (s, 2H); ^13^C NMR (CDCl_3_, 100 MHz) δ 12.5, 17.5, 23.5, 117.1, 119.3, 138.5, 155.6.

### 2,6-Dimethyl-4-*O*-triisopropylsilylbenzaldehyde (3)

To a solution of **2 **(2.00 g, 5.6. mmol) in tetrahydrofuran (10 mL) at -78°C under inert atmosphere was added of *n*-butyllithium (3.0 mL, 2.0 M in pentane). The reaction mixture was stirred for 30 min at -78°C and then DMF (0.82 g, 11.2 mmol) was added. The reaction mixture was stirred for 1 h at -78°C and for 1 h at room temperature, diluted with ethyl ether (10 mL), washed with 10 mL of water acidified with 1 M HCl, and brine (5 × 10 mL). The organic layer was dried over anhydrous MgSO_4_, filtered, and evaporated to give the crude product, which was purified by column chromatography through silica gel, using *n*-hexane:ethyl acetate (90:10) as eluent, to produce **3 **(1.20 g, 3.93 mmol, 70%) as a clear oil. ^1^H NMR (CDCl_3_, 400 MHz) δ 1.12 (d, 18H, J = 6.9 Hz), 1.25 (m, 3H), 2.55 (s, 6H), 6.55 (s, 2H), 10.50 (s, 1H); ^13^C NMR (CDCl_3_, 100 MHz) δ 12.5, 17.6, 20.7, 119.3, 120.5, 144.5, 159.6, 191.5.

### 3,5-Dimethyl-4-(3'-isopropyl-4'-*O*-methoxymethylbenzylhydroxy)-*O*-triisopropylsilylphenol (4)

*n*-butyllithium (2,90 mL, 2.0 M in pentane) was added to a solution of **1 **(1.00 g, 3.8 mmol) in tetrahydrofuran (10 mL) at -78°C under inert atmosphere. The reaction mixture was stirred for 30 min at -78°C and then aldehyde **3 **(1.18 g, 3.9 mmol) in tetrahydrofuran (10 mL) was added. The mixture was stirred for 1 h at -78°C and for 6 h at room temperature. After that, the reaction mixture was diluted with ethyl ether (10 mL), washed with 20 mL of water acidified with 1 M HCl, and brine (5 × 10 mL). The organic layer was dried over anhydrous MgSO_4_, filtered, and evaporated to give the crude product, which was purified by chromatography through silica gel, using *n*-hexane:ethyl acetate (90:10) as eluent, to yield **4 **(1.07 g, 2.15 mmol, 57%) as an oil. ^1^H NMR (CDCl_3_, 400 MHz) δ 1.10 (d, 18H, *J *= 6.9 Hz), 1.20 (dd, 6H, *J *= 6.6, 6.9 Hz), 1.25 (m, 3H), 2.24 (s, 6H), 3.35 (heptet, 1H, *J *= 6.9 Hz), 3.51 (s, 3H), 5.22 (s, 2H), 6.25 (s, 1H), 6.62 (s, 2H), 6.98 (m, 2H), 7.20 (s, 1H); ^13^C NMR (CDCl_3_, 300 MHz) δ 12.5, 17.5, 20.6, 22.5, 27.0, 31.5, 56.0, 71.0, 94.5, 113.5, 120.0 123.5, 132.0, 136.1, 137.0, 138.2, 153.0, 155.0.

### 3,5-Dimethyl-4-(3'-isopropyl-4'-*O*-methoxymethylbenzyl)-*O*-triisopropylsilylphenol (5)

A solution of **4 **(0.73 g, 1.50 mmol) in methanol (5 mL) was hydrogenated using 10% palladium on activated carbon powder (50 mg) under 2 atm of hydrogen at room temperature. After 12 h of reaction, the catalyst was removed by filtration and the solvent was evaporated under reduced pressure, to furnish the crude compound **5 **(0.565 g, 1.2 mmol) as an oil, that was used in the next step without further purification. ^1^H NMR (CDCl_3_, 400 MHz) δ 1.10 (d, 18H, *J *= 6.9 Hz), 1.20 (d, 6H, *J *= 6.9 Hz), 1.30 (m, 3H), 2.15 (s, 6H), 3.30 (heptet, 1H, *J *= 6.9 Hz), 3.50 (s, 3H), 3.90 (s, 2H), 5.15 (s, 2H), 6.60 (s, 2H), 6.67 (dd, 1H, *J *= 2.4 Hz, 8.4 Hz), 6.90 (m, 2H); ^13^C NMR (CDCl_3_, 100 MHz) δ 12.5, 17.0, 20.5, 22.8, 27.0, 33.8, 56.0, 95.0, 114.0, 119.5, 125.5, 130.0, 133.5, 137.5, 138.0, 152.5, 154.0.

### 3,5-Dimethyl-4-(3'-isopropyl-4'-*O*-methoxymethylbenzyl) phenol (6)

The crude compound **5 **(0.40 g, 0.85 mmol) and tetra-*n*-butyl-ammonium fluoride (1.06 mmol, 1.0 M in tetrahydrofuran) were stirred to provide the selective removal of the silyl proteting group. After 10 min, the reaction mixture was diluted with ethyl acetate (10 mL) and washed with brine (3 × 5 mL), dried over anhydrous MgSO_4_, filtered, and concentrated. The crude product was purified by column chromatography through silica gel, using *n*-hexane:ethyl acetate (80:20) as eluent, to yield **6 **(0.25 g, 0.79 mmol, 93% from **4**); ^1^H NMR (CDCl_3_, 400 MHz) δ 1.20 (d, 6H, *J *= 6.9 Hz), 2.15 (s, 6H), 3.30 (heptet, 1H, *J *= 6.9 Hz), 3.48 (s, 3H), 3.91 (s, 2H), 5.15 (s, 2H), 6.60 (s, 2H), 6.67 (dd, 1H, *J *= 2.4, 8.4 Hz), 6.90 (d, 1H, *J *= 8.4 Hz), 6.95 (d, 1H, *J *= 2.4 Hz); ^13^C NMR (CDCl_3_, 100 MHz) δ 20.5, 23.0, 27.2, 34.0, 56.5, 95.0, 114.5, 115.0, 125.5, 126.0, 130.0, 133.5, 137.5, 139.0, 153.5.

### GC-1 [3,5-Dimethyl-4-(4'-hydroxy-3'-isopropylbenzyl) phenoxy] acetic acid (7)

To a slurry of NaH (0.049 g, 1.02 mmol) in tetrahydrofuran (10 mL) at reflux was added compound **6 **(0.25 g, 0.79 mmol) and after 30 min a solution of bromoacetic acid (0.074 g, 053 mmol) in tetrahydrofuran (10 mL). The reaction mixture was stirred for 4 h at reflux, poured into 2 mL of cold 1 M HCl, and extracted with ethyl acetate (3 × 10 mL). The combined organic portions were dried over anhydrous MgSO_4_, filtrated, and evaporated. The residue was diluted with methanol (10 mL) and 3 drops of 6 M HCl was added. The reaction mixture was stirring for 24 h at room temperature, and the solvent was evaporated. The crude product was purified by column chromatography through silica gel, using *n*-hexane:ethyl acetate (90:10) as eluent, to yield **GC-1 **(0.19 g, 0.58 mmol, 73%). ^1^H NMR (CDCl_3_, 400 MHz) δ 1.15 (d, 6H, J = 7.1 Hz), 2.15 (s, 6H), 3.10 (heptet, 1H, J = 6.8 Hz), 3.86 (s, 2H), 4.60 (s, 2H), 6.49–6.62 (m, 2H), 6.65 (s, 2H), 6.85 (s, 1H); ^13^C NMR (CDCl_3_, 100 MHz) δ 21.0, 23.3, 28.0, 34.5, 68.5, 115.5, 116.0, 126.0, 126.5, 126.8, 131.9, 135.8, 139.4, 153.0, 157.3, 178.1.

### Protein expression and purification

The TRα-LBD and TRβ-LBD constructs were fused in frame to the C-terminus of a poly-histidine (his) tag into a pET28a(+) plasmid (Novagen).

**hTRα **The human TRα1 LBD construct including amino-acid residues Glu148-Val410 (NCBI protein accession No. A40917) was expressed in *Escherichia coli *strain B834 (Novagen). The expression and purification of the GC-1 bound TRα-LBD was accomplished as described in Nunes *et al. *[36].

**hTRβ **The human TRβ1-LBD construct, which includes amino-acid residues Glu202-Asp461 (NCBI protein accession No. NP000452), was expressed in *E. coli *strain BL21(DE3) (Stratagene). A Luria Broth (LB) starter culture was inoculated with a single colony of a LB-agar culture and grown overnight at 37°C. The initial culture was inoculated at 1% in a major 2× LB culture and grown at 22°C in kanamycin medium until the A600 nm reached 1.5. Then 0.5 mM isopropylthio-β-D-galactoside (IPTG) was added and the culture was incubated for 4–6 hours at 22°C. The induced cultures were harvested by centrifugation and the pellets were resuspended in 50 mM Tris-HCl, pH 8.0, 150 mM NaCl, 0.05% Tween 20, 20 mM β-mercaptoethanol. Phenylmethylsulfonylfluoride (PMSF) and lysozyme were added to 1 mM and 250 μg/ml, respectively, and the culture was placed on ice for 30 min. The lysate was sonicated and clarified by centrifugation for 20 minutes at 14.000 rpm in a Sorvall SS34 rotor at 4°C. To produce the holo protein, GC-1 ligand was added right after supernatant clarification in a molar excess of 10× and incubated for 1 hour at 4°C. The supernatant was incubated in batch with 1 ml Talon Superflow Metal Affinity Resin (Clontech)/liter of culture for 1 hour at 4°C. The resin was washed with 50 mM Sodium Phosphate, pH 8.0, 300 mM NaCl, 10% glycerol, 0.05% Tween 20, 10 mM β-mercaptoethanol. The bound TRβ protein was eluted with 50 mM Sodium Phosphate, pH 8.0, 300 mM NaCl, 10% glycerol, 0.05% Tween 20, 10 mM β-mercaptoethanol, 500 mM imidazol in a single step. After this step the protein was loaded into the gel filtration column HL Superdex 75 26/60 (Amersham Bioscience) equilibrated with 20 mM HEPES, 1 mM EDTA, 3 mM DTT, 0.01% Tween 20, 200 mM NaCl. The protein recovered was concentrated by ultra filtration (Amicon Ultra 10 MWCO, Millipore).

Protein content and purity of all chromatographic fractions were checked by Coomassie Blue stained sodium dodecyl sulfate-polyacrylamide gel elestrophoresis (SDS-PAGE). The average yield of the protein, with purity higher than 95%, is 15–20 mg per liter of culture. Protein concentrations were determined using the Bradford dye assay (Bio-Rad) and bovine serum albumin as standard.

### Crystallization and x-ray analysis

Crystals of the complexes TRα and TRβ were grown by hanging-drop vapour diffusion. The crystals were grown at 277 and 291 K by the sparse-matrix method, using the macromolecular crystallization reagent kits I and II (Hampton Research). In each trial, a hanging drop of 1–3 μl of protein solution (10 mg/ml in water), containing GC-1 or T3, was mixed with 1–3 μl of precipitant solution and equilibrated against a reservoir containing 500 μl of precipitant solution. For both TRα and TRβ complexes, further optimization at 291 K led to crystallization conditions similar to those reported for human TRβ LBD [16].

Crystals of TRα LBD grew within 12–24 hours from a mixture of the protein construct at 10 mg/ml mixed with the reservoir solution containing 1.0 M to 1.2 M sodium acetate and 100 mM sodium cacodilate, pH 7.2 in 1:1 proportion. Two crystal forms has been observed in a nearly identical crystallization conditions: one belonging to the orthorhombic space group P2_1_2_1_2_1 _and another – to a monoclinic space group C2.

TRβ LBD crystals grew from a 1:1 mixture of protein at the concentration of 10 mg/ml with the reservoir solution containing 1.2 M sodium acetate, 200 mM sodium succinate and 100 mM sodium cacodilate, pH 7.5 within 12–24 hours. The crystals space group was P3_1_21.

X-ray data collection was done with a MAR Research MAR345dtb image-plate detector mounted on a Rigaku ultraX 18 rotating anode X-ray generator, equipped with an OSMIC confocal Max-Flux optics and operated at 50 kV and 100 mA. To prevent radiation damage, crystals were briefly soaked in a cryoprotectant solution containing 20% and 15% (v/v) ethylene glycol, respectively for TRα and TRβ, and rapidly cooled in a gaseous nitrogen stream (Oxford Cryosystems) at 100 K. In all cases, the oscillation range was 1°, with exposure times of 15 min per image. A single data set was collected for each crystal. The data sets were reduced, merged, integrated and scaled using the softwares DENZO and SCALEPACK [38]. A summary of the data processing is given in Table 1.

### Structure Determination and Refinement

The structures were determined by molecular replacement using the software AMORE and the TRβ LBD+T3 (PDB code: 1BSX) structure as a model[39]. We have alternately run cycles of refinement using REFMAC5, and model building using O and COOT [40-42]. hTRβ electron density is fragmented in the region between residues Ala253 and Lys263, which could not be modelled. For hTRα+GC-1 complex in P2_1_2_1_2_1 _(the first crystal form) the final model consists of residues 144–410 plus 477 water molecules and a molecule of GC-1, while the hTRα+GC-1 structure in C_2 _(the second crystal form), the model consists of residues 144–405 and one GC-1 molecule. Because of the higher resolution and better quality of the X-ray data, we based our structural comparison mostly on the hTRα model refine in the first crystal form, unless explicitly stated. hTRβ+GC1 complex contains residues 201–252 and 264–460, 36 waters and one ligand molecule. hTRα+T3 model contains residues 142–408, 427 water molecules and a molecule of T3, and hTRβ+T3 structure consists of residues 202–252 and 264–460, 128 water molecules and one ligand molecule.

### Cell culture, electroporation, and luciferase cell-based assays

HeLa cells were cultured in DMEM media, containing 10% fetal bovine serum, 2 mM glutamine, 50 units/ml penicillin, and 50 μg/ml streptomycin. Cells were collected and resuspended in phosphate-buffered saline containing 0.1% dextrose and 0.01% Ca^2+^. For each transfection, 1 μg of GAL•TR expression vector (GAL4 DBD fused to hTRβ 1 LBD) was cotransfected with 4 μg of the reporter gene contained five GAL binding sites upstream of the adenovirus E1b minimal promoter linked to luciferase coding sequence (LUC). Cells were electroporated at 300 mV and 950 microfarads, transferred to fresh DMEM media, and then distributed in 12-well plates. After incubation for 20 h at 37°C and 5% CO_2_, with ethanol, or 1 μM T3, or 1 μM GC-1, the cells were collected and the pellets were solubilized by addition of 150 μl of 100 mM Tris-HCl, pH 7.8 containing 0.1% Triton X-100. The LUC activity was analyzed by adding 25 μl of luciferin to 25 μl of the lysate immediately before measurements in a luminometer (Luciferase Assay System, Promega).

### Molecular dynamics simulations

Coordinates for TRa LBD and TRβ LBD structures, obtained as described above, were used in the molecular dynamics simulations. Missing residues were modeled, particularly the O-loop in TRβ structures. The modeling was performed by finding the lowest energy structure that contain the missing residues for which the N-terminal and C-terminal ends fit the restraints of the positions they had to have in order to be well incorporated into the overall LBD structure. The complete structures were then solvated by a water shell of at least 15 Å around the LBD using with the package Packmol [43]. Sodium and chloride ions were also added to solvent to keep the overall systems neutral. No periodic boundary conditions were used. These solvated systems were then minimized by 1000 steps of conjugate-gradient minimization as implemented in NAMD keeping, however, all the protein and ligand atoms, except the modeled ones, fixed [44]. Subsequently, 100 ps of thermalization at 298.15 K with velocity scaling at every 1 ps was performed, again keeping all atoms, except the modeled ones, fixed. Following these first steps of thermalization, another 100 ps of simulations were performed with velocity scaling at every 1 ps, but keeping only the α-carbon atoms of non-modeled residues fixed. Finally, 100 ps of thermalization with velocity scaling at every 1 ps were performed without any position restraint. From this final structure, unrestrained simulations 1 ns long were performed in the NVE ensemble and the interaction energies were computed from these last simulations. The systems were composed by approximately 54,000 atoms each. In all simulations CHARMM parameters were used. Ligand parameters were obtained by group analogy from the CHARMM set, and their charges were computed as described previously [20]. All parameters are available in Martínez *et al. *[21]. In our simulations all Arg, Lys, Glu and Asp residues were considered charged. The binding pocket contains three nearby Arg residues which could eventually be deprotonated due to electrostatic repulsions involved. The presence of the ligand's carboxylate, the basicity, and the proximity to the solvent of these residues led us to choose to simulate them charged, although some alternative protonation states cannot be ruled out. In these and previous simulations such choice resulted in satisfactory representations of the mobility of the binding pocket residues [20,21]. The interaction energies reported are averages obtained from the energies computed for each individual simulation snapshot considered as representatives of each binding mode. The RMSD reported in Figure 4(a) is an indicative only of the internal flexibility of the Arg residue since it was computed after a rigid-body alignment of this residue to its conformation in the first frame of the simulations.

## Authors' contributions

PW, JDB, FARN, LAS, MSS and IP conceived the study. LB solved the crystal structure of GC-1 bound to hTRβ, carried out the structural analysis from crystallographic models and wrote the article main sessions (descriptions of materials and methods and technique-specific results, i.e., GC-1 synthesis, protein expression and purification, crystallization luciferase based assays and molecular dynamics were written by the authors who carried out each experiment, as indicated hereafter). RA and IP solved the crystal structures of GC-1 bound to hTRα and developed the initial model of ligand binding for this isoform. FMN crystallized the hTRα+GC-1 and hTRα+T3 complexes. LM did the molecular dynamics simulations. LM and MSS analyzed the MD results and also contributed to the interpretation of the structural data and to the writing of the article. SMGD developed the protocols for expression and purification, and carried out the crystallization experiments for the β isoform along with ACMF. MAMS did the experiments for cell culture, electroporation and luciferase cell-based assays. WHV, RS and PMD synthesized GC-1 samples. PW, JDB, FARN, MSS and IP interpreted the data and wrote the paper. All authors read and approved the final manuscript.

## Supplementary Material

Additional file 1 Microsoft word file containing the luciferase assays data and the synthetic route for GC-1. Click here for file
