# Supplementary material for: Structural basis of GC-1 selectivity for thyroid hormone receptor isoforms
Source: BMC Struct Biol. 2008 Jan 31;8:8. doi: 10.1186/1472-6807-8-8 (PMC2275733; doi:10.1186/1472-6807-8-8)
Supplement: Additional file 1 — Microsoft word file containing the luciferase assays data and the synthetic route for GC-1. [file 1472-6807-8-8-S1.PDF]

“Structural basis of GC-1 selectivity for thyroid hormone receptor isoforms”

Supplementary Data

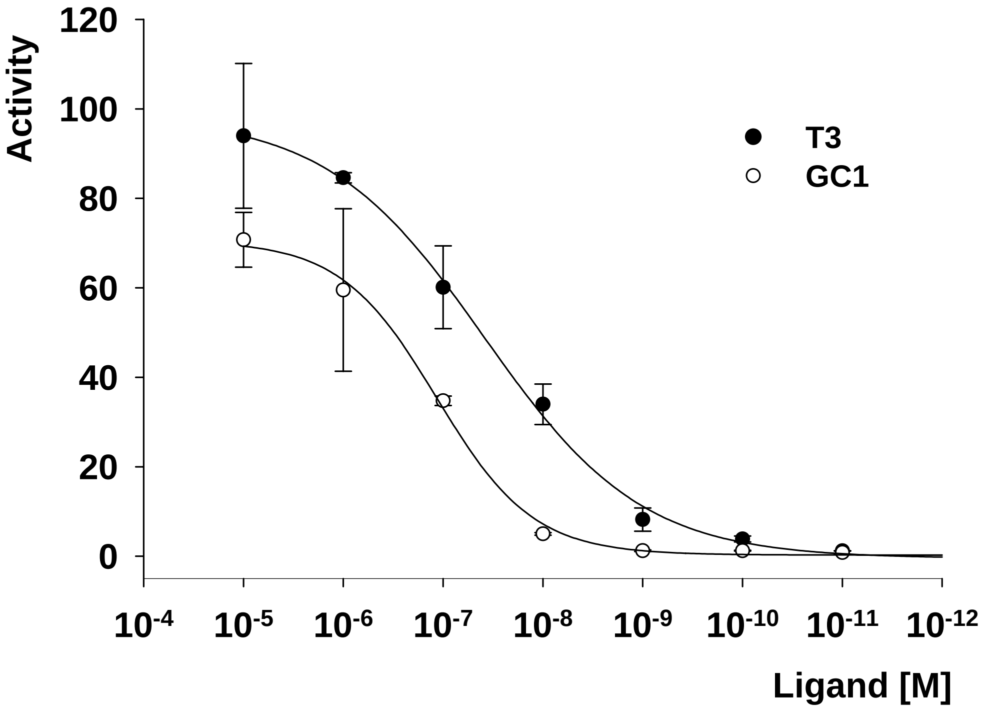

Fig. 1S: Fold activation of the transcription of luciferase by T3 and GC-1 plotted versus concentration of each ligand. Luciferase activities were measured in cell extracts as described (Materials and Methods).

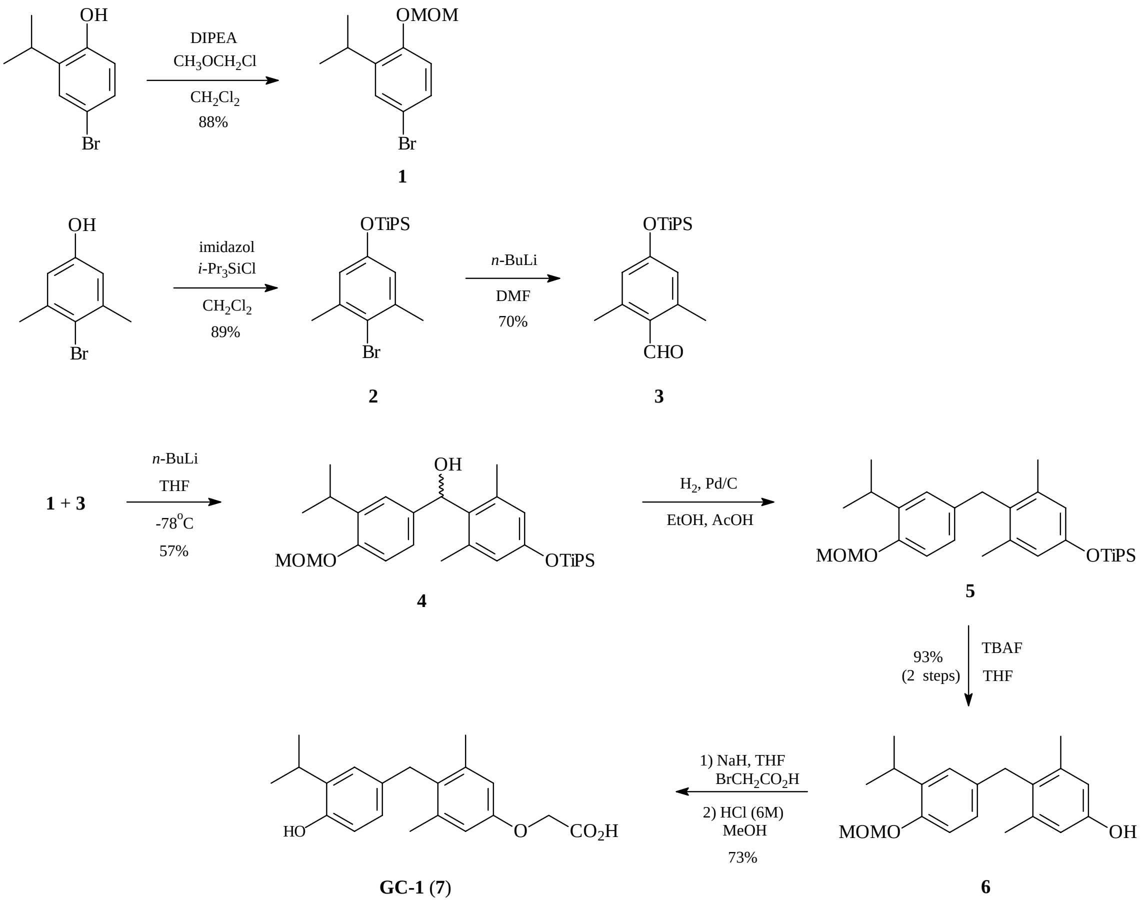

Fig. 2S: Synthetic route of GC-1.
